# Medial Orbitofrontal Cortex Regulates Instrumental Conditioned Punishment, but not Pavlovian Conditioned Fear

**DOI:** 10.1093/texcom/tgaa039

**Published:** 2020-07-30

**Authors:** Cassandra Ma, Philip Jean-Richard-dit-Bressel, Stephanie Roughley, Bryce Vissel, Bernard W Balleine, Simon Killcross, Laura A Bradfield

**Affiliations:** 1 School of Psychology, University of New South Wales, Sydney, NSW 2052, Australia; 2 Centre for Neuroscience and Regenerative Medicine, Faculty of Science, University of Technology Sydney, Sydney, NSW 2007, Australia; 3 St. Vincent’s Centre for Applied Medical Research, St. Vincent’s Hospital Sydney Limited, Darlinghurst, Sydney, NSW 2010, Australia

**Keywords:** conditioned punishment, medial orbitofrontal cortex, passive avoidance, Pavlovian conditioned fear

## Abstract

Bidirectionally aberrant medial orbitofrontal cortical (mOFC) activity has been consistently linked with compulsive disorders and related behaviors. Although rodent studies have established a causal link between mOFC excitation and compulsive-like actions, no such link has been made with mOFC inhibition. Here, we use excitotoxic lesions of mOFC to investigate its role in sensitivity to punishment; a core characteristic of many compulsive disorders. In our first experiment, we demonstrated that mOFC lesions prevented rats from learning to avoid a lever that was punished with a stimulus that coterminated with footshock. Our second experiment demonstrated that retrieval of punishment learning is also somewhat mOFC-dependent, as lesions prevented the extended retrieval of punishment contingencies relative to shams. In contrast, mOFC lesions did not prevent rats from reacquiring the ability to avoid a punished lever when it was learned prior to lesions being administered. In both experiments, Pavlovian fear conditioning to the stimulus was intact for all animals. Together, these results reveal that the mOFC regulates punishment learning and retrieval in a manner that is separate from any role in Pavlovian fear conditioning. These results imply that aberrant mOFC activity may contribute to the punishment insensitivity that is observed across multiple compulsive disorders.

## Introduction

There are relatively few studies of medial orbitofrontal cortex (mOFC) function in rodents, despite its homologous region (i.e., mOFC, which can comprise anything from the more general ventral medial prefrontal cortex to the more specific Brodman’s Area 13) being of intense interest in human studies. This interest has arisen, in part, because aberrant activity in mOFC has been consistently identified in individuals with compulsive disorders, such as substance use disorder and obsessive–compulsive disorder (OCD) (Fineberg et al. 2011; Fettes et al. 2017; Moorman 2018). Thus, it is somewhat surprising that researchers have not made better use of rodent studies in order to make precise, causal inferences about the cognitive and behavioral consequences of dysregulated mOFC activity.

One prominent rodent study that has provided causal evidence of a link between aberrant mOFC activity and compulsive actions was that by Ahmari et al. (2013), who demonstrated that the aberrant excitation (via optogenetics) of mOFC terminals in ventral striatum can generate OCD-like grooming in mice. However, the nature of mOFC aberrance in compulsive individuals is complex, and is not limited to excitation but also features abnormal inhibition (Remijnse et al. 2006; Moorman 2018). Moreover, although compulsive-like grooming is a useful measure of repetitive behavior (Kalueff et al. 2016), compulsive disorders are highly heterogeneous (Brady and Sinha 2005; Mataix-Cols et al. 2005) and grooming likely represents only a subset of related behaviors. Therefore, it is the aim of the current study to determine whether inactivating the mOFC produces another facet of compulsive disorders: insensitivity to punishment.

The inability to avoid punishing actions is a core characteristic of compulsive disorders (Hester et al. 2013; Figee et al. 2016). An individual with substance use disorder, for example, might persist in drug-seeking and drug-taking behaviors despite adverse effects on their relationships, health, and finances. Likewise, an individual with OCD might continue to wash their hands despite developing painful sores. If aberrant mOFC activity is found to cause insensitivity to punishment, this could reveal why this brain region is consistently implicated across compulsive disorders that share this core characteristic, despite otherwise varying widely in other ways. It is worth noting that such “clinical” implications of the current study should be approached with caution, particularly as the question of homology between human, primate, and rodent OFC remains somewhat contentious (Rolls and Grabenhorst 2008; Laubach et al. 2018). Nevertheless, several recent circuit-based (Heilbronner et al. 2016) and function-based (Wallis 2012; Bradfield and Hart 2020) comparisons support the notion that there is at least some degree of functional, anatomical, and circuit-based homology between species.

Insight into why aberrant mOFC inhibition might lead to compulsive-like actions can be garnered from our prior rat work (Bradfield et al. 2015, 2018). Specifically, we found that inactivating mOFC prevented rats from inferring the consequences of their actions, particularly when those outcomes are unobservable. Although this work was done using appetitive outcomes (in this case, food pellets), if an intact mOFC is also necessary to infer punishing outcomes such as footshock then inactivating it should prevent animals from learning to avoid the action that led to that footshock. This was the hypothesis that was tested in the current study. We further hypothesized, in line with prior findings (Bradfield et al. 2015; Bradfield and Hart 2020), that this role for mOFC would be specific to avoidance of the punished action, and that mOFC lesions would not affect the acquisition or expression of Pavlovian conditioned fear to footshock-associated stimuli. To test these hypotheses, we compared animals with sham and excitotoxic lesions of the mOFC on several variants of a conditioned punishment procedure (Killcross et al. 1997), as this is a unique procedure which allows for a fully dissociated assessment of punishment-driven (instrumental/action) and fear-driven (Pavlovian/stimuli) suppression of responding.

## Materials and Methods

### Experiment 1: Medial Orbitofrontal Cortex Regulation of Conditioned Punishment Learning

#### Subjects

The subjects were experimentally naïve female and male Long-Evans rats (*N* = 32, female = 16, male = 16) supplied by the University of New South Wales (Sydney, NSW, Australia). Animals were 15–20 weeks old, and female rats weighed between 220 and 310 g, and male rats weighed between 390 and 530 g, at the beginning of the experiment. Rats were housed in home cages in groups of four in a temperature- and humidity-controlled room on a 12-h light/dark cycle (lights on at 0700). Experiments were conducted during the light cycle. During behavioral training and testing, rats were maintained at ~85% of their free-feeding body weight and were allowed access to water ad libitum. All procedures were approved by the UNSW Animal Ethics Committee and are in accordance with the code outlined by the National Health and Medical Research Council (NHMRC) of Australia for the treatment of animals in research.

#### Apparatus

Behavioral procedures were conducted in standard operant conditioning chambers (Med Associates, Inc.), and controlled and recorded using Med-PC IV computer software (Med Associates, Inc.). A pellet magazine was base-centered on the right end wall, with one retractable lever on either side. Pellets (Bioserv, Biotechnologies) were delivered from a dispenser connected to the magazine. An infrared light situated at the magazine opening was used to detect head entries. A 10-s 3 kHz tone or 10-s 5 Hz flashing light were used as CSs, and 0.5-s footshocks with intensity ranging from 0.3 to 0.6 milliamps (mA) were delivered to the grid floor from a constant-current generator (see conditioned punishment procedure below for details). A house light was top-centered on the left end wall, and was illuminated throughout the entirety of each behavioral session.

#### Behavioral Procedures

##### Magazine Training

Rats initially received one session of magazine training, during which both levers were retracted and pellets were delivered to the food magazine on a variable interval-60 (VI-60s) schedule. Magazine training was terminated and the rat removed from the operant chamber once either 20 pellets had been delivered or 30 min had elapsed, whichever came first.

##### Lever Press Training

Following magazine training, rats received 12 days of lever press training for pellets. On days 1–2, rats received two 30-min sessions, one on the left lever and one on the right (order counterbalanced). For these sessions, lever presses were continually reinforced (i.e., every lever press earned a pellet). After 20 pellets were delivered or 30 min had elapsed, whichever came first, the session was terminated, levers retracted, and house lights turned off. Lever training on days 3–6 was identical (one lever per day, order counterbalanced), except rats could earn as many pellets as the schedule allowed. Pellets were delivered on a variable interval schedule averaging 15 s (VI-15s) on days 3–4, which was increased to a VI-30s schedule on days 5–6.

Rats were then trained to press both levers (i.e., both levers were available throughout the entire session) on days 7–12 during 30 min sessions. On day 7, responding on each lever was rewarded on a VI-15s schedule. On days 8–12, responding was reinforced on a VI-30s schedule. Lever press training on days 8–11 was intended to equalize responding on each lever and remove any biases animals might show in responding on the left versus right lever. To achieve this, a modified VI-30s schedule was implemented in which pellets were more frequently available on the nonpreferred lever and less frequently available for delivery on the preferred lever (VI schedule adjusted as a ratio of responding on each lever). The last day (day 12) consisted of an unmodified VI-30s schedule on both levers to obtain an unbiased measure of prepunishment lever pressing rates. Immediately after the last lever press training session, animals were returned to ad libitum access to chow in their home cages for 3 days prior to surgeries being carried out (see section on surgeries below for details).

##### Conditioned Punishment

Following at least 7 days of recovery from surgeries, rats were again placed on food restriction as described. After 3 days of food restriction, rats received 5 days of conditioned punishment training. Each conditioned punishment training session lasted 60 min during which both levers were presented throughout, and both continued to earn pellets on a VI-30s schedule as they had previously. In addition, the punished lever earned an aversive CS+ that coterminated with footshock on a VI-60s schedule, whilst the unpunished lever earned a neutral CS– that terminated without consequence (also on a VI-60s schedule).

The experiment was fully counterbalanced such that, for half of the animals, the CS+ was the tone and the CS− was the flashing light, and for the other half, the CS+ was the flashing light and the CS− was the tone. The left lever was punished (i.e., earned CS+ and footshock) and the right lever was unpunished (i.e., earned the CS−) for half of the animals, whereas the other half received the opposite arrangement. The 0.5-s footshock that coterminated with the CS+ increased in intensity over days. Daily foot-shock schedules over the 5 days were 0.3, 0.4, 0.4, 0.5, 0.5 mA for males, and 0.3, 0.3, 0.4, 0.4, 0.5 mA for females. If a lever press was scheduled to deliver both a pellet and a CS at the same time, only the CS +/− footshock was delivered due to its leaner schedule.

Punishment avoidance was determined as fewer lever presses on the punished versus the unpunished lever when no stimuli were present (i.e., during the intertrial interval; ITI). Suppression of responding on both levers during the aversive CS+ (10 s per presentation) was recorded and used to calculate a conditioned suppression ratio as a measure of Pavlovian fear (see Statistical Analysis for details).

#### VI-30 Conditioned Punishment

Following conditioned punishment training, rats completed another 5 days of conditioned punishment training with the reinforcement schedules altered such that the pellets and CSs were earned at double the frequency of initial punishment training. Specifically, pellet delivery was increased to a VI-15s schedule (previously VI-30s) and CS presentation was increased to a VI-30s schedule (previously VI-60s). The duration of the CS presentations remained at 10 s.

#### Surgery

It should be noted that we initially proposed to investigate the differences between anterior and posterior lesions of the mOFC in conditioned punishment, although these groups were eventually collapsed across (see results). Thus, separate anterior and posterior co-ordinates are described.

Stereotaxic surgery was conducted under isoflurane anesthesia (5% induction; 1–2% maintenance). Each rat was placed in a stereotaxic frame (David Kopf Instruments, Tujunga, CA), after which they received a 0.1 mL subcutaneous injection of bupivacaine hydrochloride at the incision site. An incision was made into the scalp to expose the skull surface, and the incisor bar was adjusted to place bregma and lambda in the same horizontal plane. Anterior mOFC co-ordinates were (in mm relative to bregma): +5 anteroposterior, ±0.6 (males) or ±0.5 (females) mediolateral, and −4.6 dorsoventral. Posterior mOFC co-ordinates were (in mm relative to bregma): +3.8 anteroposterior, ±0.7 (males) or ±0.6 (females) mediolateral, −5.7 dorsoventral.

Excitotoxic lesions were made by infusing 0.3 μL of N-methyl-D-aspartate (NMDA: 10 mg/mL) in sterilized 0.1 M phosphate buffered saline (PBS) pH 7.2 over 3 min. The needle was left in place for 2 min prior to removal to allow for diffusion. Sham-operated rats underwent the same procedures but no excitotoxin was infused. Half of the sham lesions were performed at the anterior coordinates and the other half at the posterior coordinates. All rats received a subcutaneous injection of 0.1 mL carprofen and a 0.4 mL intraperitoneal injection of procaine penicillin solution (300 mg/kg). Rats were given 7 days to recover from surgery, after which they received 3 days of food deprivation prior to the commencement of experimentation. Rats were weighed and handled daily during this time.

#### Tissue Preparation for Histological Analysis

Animals received a lethal dose of sodium pentobarbital (300 mg/kg i.p., Virbac Pty. Ltd, Australia) 1–2 days following the completion of behavioral testing. Their brains were extracted and stored in a refrigerator kept at −30 °C. They were then frozen on Optimum Cutting Temperature (OCT) compound (Tissue-Tek, Sakura Finetek) and coronally sectioned at 40 μm through the mOFC using a cryostat (Leica CM1950, Leica Biosystems) maintained at approximately −18 °C.

Each section of the mOFC was collected directly onto a slide and was stained with NeuroTrace fluorescent nissl (Invitrogen) and left to dry in darkness for at least 30 min. The slides were then cover-slipped with VectaShield (Vector Laboratories, Inc.) and left to dry overnight in darkness. Sections were examined for lesion placements on a confocal microscope by a trained observer who was naïve to group allocation. Needle track marks were also examined for all groups including group SHAM.

#### Data Analysis

Rates of responding per minute on the punished and unpunished levers during CS+, CS−, and ITI (non-CS) periods were calculated. Punishment avoidance was measured as the rate of responding on the punished versus unpunished lever during the ITI. To determine if the punishment effect differed between groups, a three-way, repeated measures ANOVA was conducted controlling the family-wise error rate at α = 0.05. For this ANOVA analysis, there was a between-subjects factor of group (i.e., Sham vs. Lesion) and a repeated measures factor of day (1–5) and of lever (punished vs. unpunished). Interactions with the “day” factor were typically nonsignificant and thus not reported, although linear trend analyses are reported as evidence of learning in some instances. If an interaction (or interactions) was detected, then follow-up simple effects analyses were calculated to determine the source of the interaction.

Pavlovian conditioned fear was measured as the amount of suppression during the CS relative to ITI. Conditioned suppression ratios were calculated separately for the CS+ and CS–. This calculation was carried according to Equation 1 for the CS+:

(1) \begin{equation*} \frac{\mathrm{All}\ \mathrm{Lever}\ \mathrm{Presses}\ \mathrm{During}\ \mathrm{CS}+}{\mathrm{All}\ \mathrm{Lever}\ \mathrm{Presses}\ \mathrm{during}\ \mathrm{ITI}+\mathrm{during}\ \mathrm{the}\ \mathrm{CS}+} \end{equation*}

And according to Equation (2) for the CS–:

(2) \begin{equation*} \frac{\mathrm{All}\ \mathrm{Lever}\ \mathrm{Presses}\ \mathrm{During}\ \mathrm{CS}-}{\mathrm{All}\ \mathrm{Lever}\ \mathrm{Presses}\ \mathrm{during}\ \mathrm{ITI}+\mathrm{during}\ \mathrm{the}\ \mathrm{CS}-} \end{equation*}

For these equations, a ratio below 0.5 indicates conditioned suppression (Pavlovian fear) to the CS, whereas a ratio of 0.5 or above indicates no fear. Once these values had been calculated separately for each CS, a three-way repeated measures ANOVA, with a between-subjects factor of group, and a repeated measures factor of day (1–5) and another of CS (CS+ vs. CS–) controlling the familywise error rate at α = 0.05, was carried out to determine whether mean conditioned suppression ratios were higher to the CS+ than to the CS− for each group. Greater suppression to the CS+ relative to CS− indicates appropriate discrimination of Pavlovian fear.

### Experiment 2: Medial Orbitofrontal Regulation of Conditioned Punishment Retrieval

Experiment 2 was conducted identically to Experiment 1, except that mOFC lesions were administered postpunishment training, and animals were subject to retrieval tests. Specifically, lever press training took place as previously described, then rats received 7 days of prelesion conditioned punishment training. Following surgical procedures and recovery, rats received a 5-min extinction test followed by a 10-min test in which only pellets were delivered. Rats were then retrained on conditioned punishment for 5 days and tested again in a 20-min test in which only pellets were delivered.

#### Subjects

Subjects were experimentally naïve female and male Long-Evans rats (*N* = 40, female = 20, male = 20) supplied by the University of New South Wales (Sydney, NSW, Australia). Animals were 15–20 weeks old, and female rats weighed between 220 and 310 g, and male rats weighed between 390 and 530 g at the beginning of the experiment. Animals were housed and maintained in identical conditions to those described for Experiment 1.

#### Behavioral Procedures

All behavioral procedures were conducted as described for Experiment 1, except rats received one less day of the modified VI-30s schedule designed to equalize lever presses on each lever, which was sufficient for rats to show an unbiased response. Further, presurgery punishment training was completed for 7 rather than 5 days to ensure both groups had developed a strong punishment effect prior to surgery. The following tests were also carried out as described below.

#### 5-min Extinction Test and 10-min Pellet-Only Test

Following recovery from surgery, rats received a 5-min test that was conducted in extinction. That is, both levers were extended and the houselight turned on, but no outcomes (pellets and CSs ± footshock) were delivered. This test was immediately followed by a 10-min pellet-only test, in which both levers were extended and responding on them earned pellets on a VI-30s schedule as during lever press training, but no CSs ± footshock were delivered.

#### Conditioned Punishment Reacquisition

Following these initial tests, rats received 5 days of retraining on conditioned punishment, which was conducted as previously described (pellets on VI-30s schedules, CSs ± footshocks on VI-60s schedules). The footshock intensity (mA) used for each rat was matched from its last day of presurgery punishment acquisition and kept consistent throughout retraining so as not to skew any behavioral effects of lesions.

#### 20-min Pellet-Only Test

Following retraining, rats received another test in which only pellets were delivered on a VI-30s schedule. This time however, the pellet-only test was not preceded by an extinction test and lasted for 20 min.

“All surgical, histological, data collection, and analyses procedures were conducted as described for Experiment 1.”

## Results

### Experiment 1: Medial Orbitofrontal Cortex Lesions Impair Conditioned Punishment Learning

Dysregulation of mOFC activity has been consistently identified in the brains of individuals with compulsive disorders such as OCD and substance use disorder (Maia et al. 2008; Fineberg et al. 2011; Moorman 2018). Insensitivity to punishment is characteristic of such disorders, yet causal evidence linking mOFC dysfunction to punishment insensitivity is lacking. We used a rodent model to examine whether lesions of mOFC caused insensitivity to punishment. To test this, we employed excitotoxic mOFC lesions in rats and assessed punishment avoidance and Pavlovian fear within a conditioned punishment procedure (Killcross et al. 1997).

In Experiment 1, we tested this hypothesis specifically with regards to conditioned punishment “learning.” Animals were first trained to press two levers (left and right) equally for pellets, after which they received sham or excitotoxic lesions of the mOFC. In the next stage of behavioral training, following recovery from surgeries, each lever continued to earn the pellets at the same rate (VI-30s), but one lever also began to occasionally earn a 10 s aversive CS+ that coterminated in mild footshock, whereas the other lever occasionally yielded a CS– that terminated without consequence. The lever that earned the CS+ was considered the punished lever, and the lever that earned the CS– was unpunished. Punishment learning was measured as avoidance of the punished lever relative to the unpunished lever during the ITIs between CS presentations, when rats’ choices were not influenced by CSs or footshocks. Rather, animals were required to infer from memory which lever would be punished and which would be unpunished. As the inference of unobservable outcomes is thought to rely on mOFC (Bradfield et al. 2015, 2018), we hypothesized that instrumental punishment learning would be impaired in group mOFC relative to group SHAM.

In addition to this instrumental punishment effect, the consistent pairing of the CS+ with footshock causes an aversive Pavlovian stimulus-footshock association to form, causing a general disruption of lever pressing on both levers (Killcross et al. 1997). This effect is known as “conditioned-suppression,” and is a well-established measure of aversive Pavlovian CS–US associations that correlates highly with freezing (Bouton and Bolles 1980). Presentation of the aversive CS+ was expected to produce conditioned suppression, whereas presentation of the neutral CS− was not. Because Pavlovian fear is measured during CS presentations (i.e., when CS’s are observable), we hypothesized that it would not rely on mOFC and would be intact for both groups.

As previously noted, it was our initial intention to compare the relative roles of anterior mOFC and posterior mOFC (posterior mOFC is the anterior section of cingulate area 32V [AV32] according to Paxinos and Watson 2014) in punishment learning, because we have previously found the anterior mOFC to be particularly important for the regulation of goal-directed action (Bradfield et al. 2018). However, for current experiments, we were unable to separate these groups either anatomically or behaviorally, so they were collapsed across for all analyses.

#### Histology

**Figure 1:**
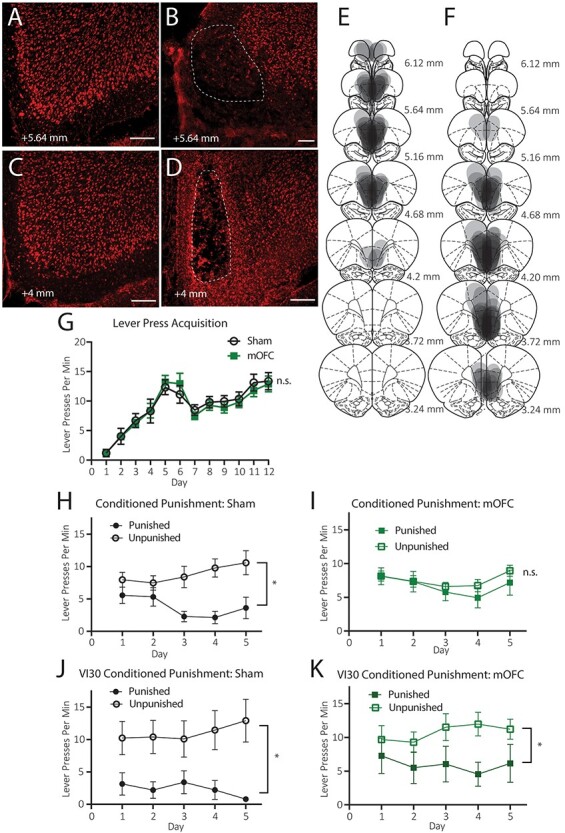
Excitotoxic lesions of medial orbitofrontal cortex prevent conditioned punishment learning. (*A*–*D*) Representative photomicrographs of (*A*) a sham anterior mOFC placement, (*B*) an excitotoxic anterior mOFC lesion (both +5.64 mm from bregma, (*C*) a sham posterior mOFC placement, and (*D*) a posterior mOFC lesion (+4 mm from bregma). Scale bars = 100 μm. (*E*) Diagrammatic representation of overlapping anterior mOFC lesion placements in Experiment 1, (*F*) diagrammatic representation of overlapping posterior mOFC lesion placements in Experiment 1, (*G*) Mean (±1 SEM) lever presses per min during initial lever press acquisition, (*H*) Mean (±1 SEM) lever presses per min during conditioned punishment training for group SHAM, (*I*) Mean (±1 SEM) lever presses per min during conditioned punishment training for group mOFC, (*J*) Mean (±1 SEM) lever presses per min during VI-30s conditioned punishment training for group SHAM, (*K*) Mean (±1 SEM) lever presses per min during VI-30S conditioned punishment training for group mOFC, note that although graphed separately, groups SHAM and mOFC groups were compared statistically (group × punishment interaction for the data displayed in Fig. 1*H–I*, *P* < 0.05, group × punishment interaction for data displayed in Fig. 1*J–K*, *P* > 0.05). ^*^P < 0.05, n.s. = nonsignificant, *P* > 0.05.

Figure 1*A* shows a sham anterior mOFC photomicrograph at +5.64 mm from bregma, and Figure 1*B* show an excitotoxic lesions of anterior mOFC at +5.64 mm from bregma. Figure 1*C* shows a sham posterior mOFC micrograph at +4 mm from bregma, and Figure 1*D* shows an excitotoxic posterior mOFC lesion at +4 mm from bregma. Figure 1*E,F* shows a representation of the overlapping lesion placements of all animals with anterior mOFC (Fig. 1*E*) and posterior mOFC (Fig. 1*F*) lesions. If greater than 25% of the cell loss from a lesion extended more than a 1 mm radius from the injection site, the rat was excluded from analysis. In total, 9 rats were excluded from Experiment 1 due to incorrect lesion placement or size, 1 rat died during surgery, and 1 rat was excluded due to a consistent and extreme preference for one lever (consistently > 100 more presses on one of the two levers) during lever press training, prior to surgeries. After exclusions, final numbers (*N* = 21) for groups in Experiment 1 were: sham control (*n* = 7), and mOFC lesions (*n* = 14: anterior *n* = 6, posterior *n* = 8). As mentioned, we collapsed across anterior and posterior mOFC lesion groups for analysis as they did not differ on any behavioral measure in either experiment (all *P* values > 0.05). Moreover, it is clear from Figure 1*E,F* that there was significant overlap between anterior and posterior lesions at approximately +4.68 mm from bregma, such that we could not confidently separate these groups anatomically. The data from Figure 1*I*,*K* are shown in accordance with animals’ initial anterior and posterior group assignments in Supplementary Figure 1*C–F*.

#### Behavior

##### Lever Press Training

######  

Lever press rates per min during initial lever press training are shown in Figure 1*G* (±SEM, averaged over levers). This training was conducted prior to lesion surgeries, however, we have shown the data in accordance with the to-be assigned groups. It is clear from this figure that groups Sham and mOFC both acquired lever press responding and did not differ in their acquisition (note that the dip in responding seen between days 6–7 resulted from the increase in response competition after animals were switched from single-lever to double-lever protocols). Indeed, there was no main effect of group, *F* < 1, but there was a linear main effect of responding over days *F*(1, 19) = 97.01, *P* = 0.00, that did not interact with group, *F* < 1.

###### Conditioned Punishment.

The mean (±SEM) lever presses per min on the punished and unpunished levers over days during conditioned punishment training (during ITIs only) are shown in Figure 1*H* for group SHAM, in Figure 1*I* for group mOFC. The same data are shown with individual data points for each animal, averaged across the 5 days of conditioned punishment training for both groups in Supplementary Figure 1*A*. From these figures, it is clear that group SHAM learned to avoid the punished lever during the ITI (unpunished > punished), but group mOFC did not (punished = unpunished). Statistical analysis showed that there was no main effect of group, *F* < 1, but there was a main effect of punishment *F*(1, 19) = 11.33, *P* = 0.003, as well as a punishment × group interaction, *F*(1, 19) = 5.023, *P* = 0.037. Follow up simple effects analysis reveal that this interaction comprised an intact punishment effect (unpunished > punished) for group SHAM, *F*(1, 19) = 11.79, *P* = 0.003, but not for group mOFC (unpunished = punished), *F* < 1. Thus, this result demonstrates that mOFC lesions prevented animals from learning conditioned punishment.

###### Punishment Learning.

During the VI-30s punishment schedule, the results suggest that mOFC lesions produce a specific deficit in instrumental conditioned punishment learning. To test how pervasive this deficit is, the same rats received five more days of conditioned punishment training with double the frequency of outcomes to determine whether this was sufficient to allow group mOFC to overcome their initial impairment. That is, CSs and footshock were now delivered on a VI-30s schedule (increased from VI-60s) and pellets were now delivered on a VI-15s schedules (increased from VI-30s to keep pellet/CS ratio consistent).

Mean (±SEM) lever presses per min on the punished and unpunished levers over days during conditioned punishment (VI-30s) training (during ITIs only) are shown in Figure 1*J* for group SHAM and in Figure 1*K* for group mOFC. The same data are shown averaged across the 5 days of conditioned punishment (VI-30s) training for both groups in Supplementary Figure 1*B*. These figures show that the increase in reinforcement rates was sufficient for group mOFC to overcome their initial impairment because they, like group SHAM, responded selectively to the unpunished lever and avoided the punished lever. This is supported by statistical analysis because here was no main effect of group, *F* < 1, but there was a main effect of punishment *F*(1, 19) = 7.54, *P* = 0.013, which this time, did not interact with group, *F* < 1.

#### Pavlovian Fear Conditioning

##### Conditioned Suppression During Initial (VI-60s) Conditioned Punishment

**Figure 2:**
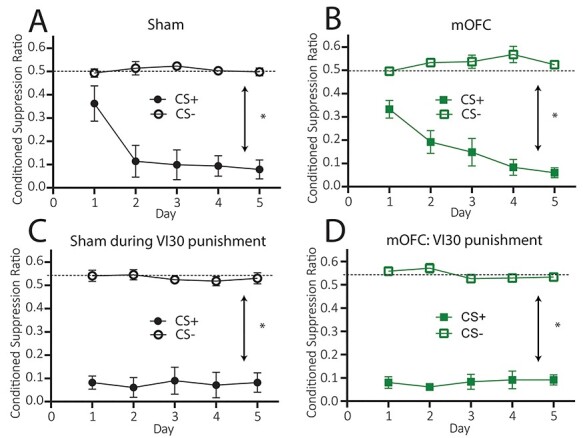
Excitotoxic lesions of medial orbitofrontal cortex leave Pavlovian fear conditioning intact. (*A*) Mean (±1 SEM) conditioned suppression ratios to CS− and CS+ for group SHAM during conditioned punishment training, (*B*) Mean (±1 SEM) conditioned suppression ratios to CS− and CS+ for group mOFC during conditioned punishment training, (*C*) Mean (±1 SEM) conditioned suppression ratios to CS− and CS+ for group SHAM during VI-30s conditioned punishment training, (*D*) Mean (±1 SEM) conditioned suppression ratios to CS− and CS+ for group mOFC during VI-30s conditioned punishment training. Note that although graphed separately, groups SHAM and mOFC were compared statistically at each stage (group × Pavlovian conditioning interaction for data displayed in Fig. 2*A*,*B*, *P* > 0.05, for data displayed in Fig. 2*C*,*D*, *P* > 0.05). ^*^P < 0.05.

Mean (±SEM) conditioned suppression ratios over days of conditioned punishment training are shown in Figure 2*A* for group SHAM and Figure 2*B* for group mOFC. It is clear that animals in both groups SHAM and mOFC suppressed lever pressing during CS+ but not during CS– presentations. Statistically, there was no main effect of group, *F* < 1, but there was a main effect of CS, *F*(1, 19) = 164.06, *P* = 0.00 that did not interact with group, *F* < 1. Thus, in contrast to instrumental punishment learning, mOFC lesions left Pavlovian fear conditioning to the CS+ intact.

##### Conditioned Suppression During VI-30s Punishment

Pavlovian fear was again unaffected during VI-30s punishment learning. Mean (±SEM) conditioned suppression ratios to the CS+ and CS– during VI-30s conditioned punishment training are shown over days in Figure 2*C* for group SHAM and Figure 2*D* for group mOFC. From these figures, it is clear that animals in both groups suppressed responding during CS+ but not CS– presentations. Statistically, there was no main effect of group, *F* < 1, but there was a main effect of CS, *F*(1, 19) = 379.72, *P* = 0.00 which did not interact with group, *F* < 1.

### Experiment 2: Medial Orbitofrontal Cortex Lesions Regulates the Retrieval but not Reacquisition of Conditioned Punishment

The results of Experiment 1 reveal a direct causal link between mOFC dysfunction and insensitivity to punishment, particularly when the punishment schedule was lean. Specifically, sham rats learned to avoid a lever that earned a CS+ coterminating in footshock, whereas rats with mOFC lesions were unable to adjust their behavior to avoid punishment. This impairment was limited to the instrumental punishment effect (i.e., unpunished > punished responding during the ITI) because mOFC lesions did not affect suppression to the CS+ (i.e., conditioned suppression during CS+ presentations was greater than suppression during the CS− for both groups). This suggests that mOFC lesions do not generally affect aversion learning, or the ability to suppress lever-pressing, but instead reveal that mOFC lesions induced a specific, punishment-insensitive phenotype identified in previous studies (Jean-Richard-dit-Bressel et al. 2019).

The effect of mOFC lesions in Experiment 1 was also limited to punishment learning, and whether mOFC is similarly necessary for the retrieval of punishment avoidance after it has been learned is unknown. This question is important because, as noted by Jean-Richard-Dit-Bressel et al. (2018), in real-world situations punishment is rarely immediate (e.g., drug withdrawals occurring the following day, financial consequences might occur months later). Thus, in order for individuals to successfully avoid punishments that may not occur until later, they must retrieve previously learned contingencies when no punishing outcomes are currently present.

To determine whether mOFC is necessary for the retrieval of punishment avoidance after it has been learned, we lesioned the mOFC after conditioned punishment training and gave animals retrieval tests in which no punishing outcomes were delivered. Specifically, following recovery from surgery, animals received a 5-min extinction test (i.e., no outcomes delivered) followed by a 10-min test in which only pellets were delivered. Because no CSs or footshocks were delivered on either test, responding in accordance with punishment contingencies required these outcomes to be inferred from memory. Thus, group mOFCs were expected to be impaired (unpunished = punished) on this test relative to group SHAM (unpunished > punished). Following testing, we retrained animals on conditioned punishment for 5 days and then tested their retrieval again in a 20-min pellet-only test.

**Figure 3:**
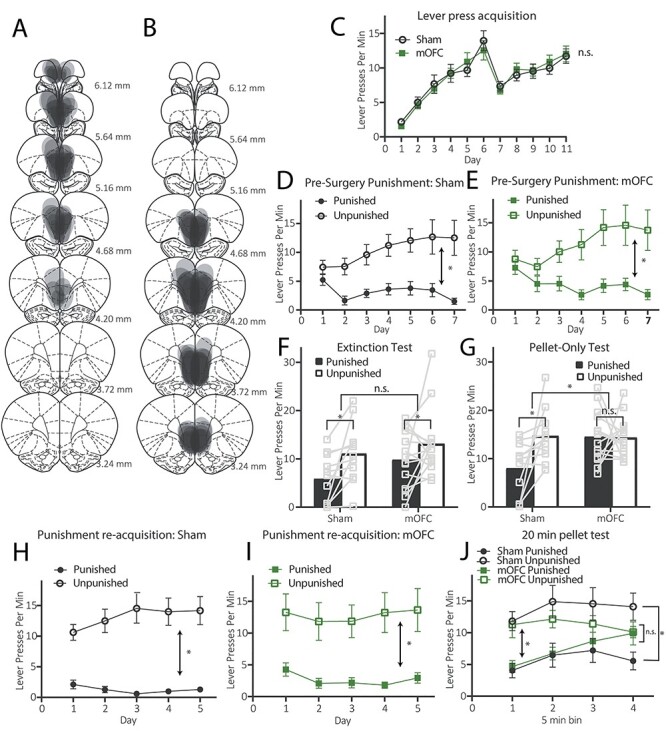
Excitotoxic lesions of medial orbitofrontal cortex impair extended conditioned punishment retrieval. (*A*) Diagrammatic representation of anterior lesion placements in Experiment 2, (*B*) diagrammatic representation of posterior lesion placements in Experiment 2, (*C*) Mean (±1 SEM) lever presses per min during initial lever press acquisition, (*D*) Mean (±1 SEM) lever presses per min during presurgery conditioned punishment training for group SHAM, (*E*) Mean (±1 SEM) lever presses per min during presurgery conditioned punishment training for group mOFC, (*F*) Mean lever presses per min for both groups during the 5-min extinction retrieval test, (*G*) Mean lever presses per min for both groups during the 10-min pellet-only retrieval test, (*H*) Mean (±1 SEM) lever presses per min during conditioned punishment retraining for group SHAM, (*I*) Mean (±1 SEM) lever presses per min during conditioned punishment retraining for group mOFC, (*J*) Mean (±1 SEM) lever presses per min during the 20-min pellet-only retrieval test, separated into 5 min bins. Note that although sometimes graphed separately, groups SHAM and mOFC were compared statistically at each stage (group × punishment interactions for data displayed in Fig. 3*D*,*E*, *P* > 0.05, for data displayed in Fig. 3*H*,*I*, *P* > 0.05). ^*^P < 0.05, n.s. = nonsignificant, *P* > 0.05**.**

#### Histology

Figure 3*A* and *B* shows a representation of the overlapping lesion placements of all animals with anterior mOFC (Fig. 3*A*) and posterior mOFC (Fig. 3*B*). If greater than 25% of the cell loss from a lesion extended more than a 1 mm radius from the injection site, the rat was excluded from analysis. Five rats were excluded from Experiment 2 due to incorrect lesion placement or size, three rats died during surgery, six rats were excluded due to malfunctioning operant conditioning chambers, and one rat was excluded due to a consistent and extreme affinity for the punished lever during conditioned punishment training prior to surgery (i.e., >100 more presses on punished than unpunished lever every day). After exclusions, final numbers (*N* = 25) for groups in Experiment 2 were: group SHAM (*n* = 11), and mOFC lesions (*N* = 14: anterior *n* = 8, posterior *n* = 6). As in Experiment 1, we collapsed anterior and posterior mOFC lesion groups for analysis as and there was significant overlap of lesions, particularly at +4.68 mm anterior to bregma, and they did not differ significantly on any measure (all *P* values > 0.05). The data from Figure 3*F*, *G*, and *J* are shown in accordance with their initial anterior and posterior group assignments in Supplementary Figure 2*A–E*.

#### Behavior

#####  

###### Lever Press Training.

Lever press rates per min during initial lever press training are shown in Figure 3*C* (±SEM, averaged over levers) shown with data split according to the groups assigned at surgery. This figure shows that the groups SHAM and mOFC both acquired lever press responding and did not differ in their acquisition (again there is a dip in responding from days 6 to 7 due to increased response competition that occurred as animals were transferred from the single-lever to the double-lever protocol). There was no main effect of group, *F* < 1, but there was a linear main effect of responding over days (1, 19) = 141.714, *P* = 0.00, that did not interact with group, *F* < 1.

###### Conditioned Punishment (Presurgery).

Conditioned punishment training occurred across 7 days prior to lesion surgeries. Data are shown according to the groups assigned during surgery. Mean (± SEM) rates of responding on the punished and unpunished levers over days (during ITIs only) are shown in Figure 3*D* for group SHAM and Figure 3*E* for group mOFC. It is clear that both groups SHAM and mOFC acquired punishment avoidance prior to lesion surgeries and did not differ in their acquisition, as both responded more on the unpunished relative than the punished lever. There was no main effect of group, *F* < 1, but there was a main effect of punishment, *F*(1, 23) = 14.97, *P* = 0.001, that did not interact with group, *F* < 1.

###### Extinction Test.

Mean (± SEM) lever presses for groups SHAM and mOFC during the 5-min extinction test are shown in Figure 3*F*. This figure shows that group SHAM responded according to the punishment contingencies (unpunished > punished). Although this effect appears to be somewhat attenuated in group mOFC, no statistical differences between the two groups were detected. Specifically, there was no main effect of group, *F*(1, 23) = 3.112, *P* = 0.091, but there was a main effect of punishment, *F*(1, 23) = 11.435, *P* = 0.003, that did not interact with group, *F*(1, 23) = 1.4, *P* = 0.25. These findings suggest that both Sham and mOFC-lesioned animals can retain and express the punishment contingencies they learned prior to the mOFC lesion, at least upon initial testing in extinction.

###### Pellet-Only Test.

Mean (±SEM) lever presses for groups SHAM and mOFC during the 10-min pellet-only test are shown in Figure 3*G*. This figures shows that group SHAM continued to respond in accordance with the punishment contingencies (unpunished > punished) but group mOFC did not (unpunished = punished). This is supported by statistical analysis, as there was a main effect of group, *F*(1, 23) = 6.13, *P* = 0.021, indicating that group mOFC responded more overall than did group SHAM, a marginal main effect of punishment, *F*(1, 23) = 3.97, *P* = 0.058, but there was a significant punishment × group interaction, *F*(1, 23) = 4.34, *P* = 0.049. Follow-up simple effects analysis showed this interaction consisted of a significant effect of punishment in group SHAM, *F*(1, 23) = 7.41, *P* = 0.012, but not group mOFC, *F* < 1. This result suggests that animals in group SHAM continued to apply the punishment contingencies they had learned presurgery (i.e., unpunished > punished) throughout the test, despite the availability of pellets and the absence of CSs and footshock. This is consistent with previous results demonstrated by Jean-Richard-Dit-Bressel and McNally (2016) in which control animals continued to apply (primary) punishment contingencies throughout the entirety of a 30-min pellet-only test. In contrast, punishment retrieval was impaired for group mOFC who responded equally on both levers.

Why mOFC lesions impaired performance on this test but not the prior extinction test is unclear. Indeed, we had predicted that mOFC lesions would impair performance on both tests due to the absence of punishing outcomes. One possibility is that group mOFC’s performance was impaired on both tests, but only the pellet test was sufficiently powered to detect a group × punishment interaction, perhaps due to more consistent responding across the 10 min which reduced variability. Another possibility is that the switch in contingencies from extinction to pellets caused confusion or interference in group mOFC that was not present in sham animals. A final possibility is that both groups initially recalled the punishment contingencies, but that their extinction was facilitated in group mOFC relative to group SHAM.

To distinguish between these possibilities, we retrained animals on the conditioned punishment contingencies for 5 days then gave them another pellet-only test, only this time the pellet-only test lasted for 20 min and was not preceded by an extinction test. Sham animals were once again expected to maintain responding in accordance with the punishment contingencies for the entire duration of the test. If mOFC lesions produce a general decrement in the retrieval of punishment contingencies which the prior extinction test was underpowered to detect, then group mOFC should be impaired for the entire 20 min. Alternatively, if group mOFCs were previously impaired due to the change in contingency from extinction to pellets, then group mOFC should display intact performance for the entire 20 min as no contingency change is applied on this test. Finally, if extinction of the punishment contingencies was facilitated in group mOFC relative to group SHAM, on the 20-min test, this group should once again initially respond according to these contingencies (unpunished > punished) then revert to equal lever pressing on both levers (unpunished = punished) for the latter part of the test.

###### Conditioned Punishment Retraining.

Mean (± SEM) lever presses per min during the reacquisition of conditioned punishment is shown over days in Figure 3*H* for group SHAM, in Figure 3*I* for group mOFC. It is clear from these figures that punishment (unpunished > punished) is intact for both groups during retraining. Statistical analysis supports this conclusion because there is no main effect of group, *F* < 1, but there is a main effect of punishment, *F*(1, 23) = 28.99, *P* = 0.00, and no punishment × group interaction, *F* < 1. This suggests that, in contrast to initial conditioned punishment learning in Experiment 1, mOFC lesions do not produce a decrement in the reacquisition of conditioned punishment.

**Figure 4:**
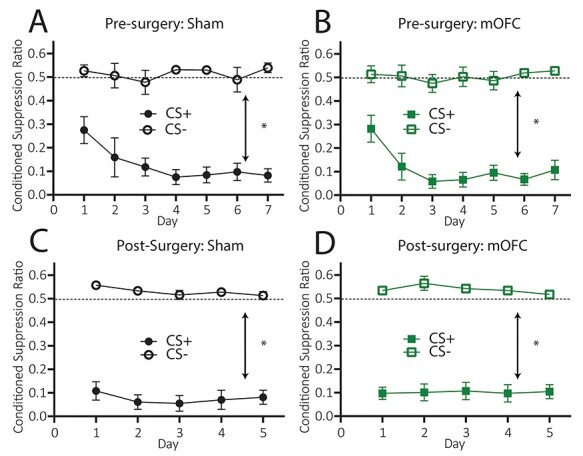
Excitotoxic lesions of medial orbitofrontal cortex leave Pavlovian fear conditioning intact. (*A*) Mean (±1 SEM) conditioned suppression ratios to CS− and CS+ for group SHAM during presurgery conditioned punishment training, (*B*) Mean (±1 SEM) conditioned suppression ratios to CS− and CS+ for group mOFC during presurgery conditioned punishment training, (*C*) Mean (±1 SEM) conditioned suppression ratios to CS− and CS+ for group SHAM during conditioned punishment retraining, (*D*) Mean (±1 SEM) conditioned suppression ratios to CS− and CS+ for group mOFC during conditioned punishment retraining. Note that although graphed separately, groups SHAM and mOFC were compared statistically at each stage (group × Pavlovian conditioning interaction for data displayed in Fig. 4*A*,*B*, *P* > 0.05, for data displayed in Fig. 4*C*,*D*, *P* > 0.05). ^*^P < 0.05.

###### 20-min Pellet-Only Test.

The question of interest was whether group mOFC would respond in accordance with the punishment contingencies (unpunished > punished) for none of the test, some of the test, or all of the test. As we had previously compared groups SHAM and mOFC in a 5-min extinction test followed by a 10-min pellet-only test, we also split the data from the 20-min pellet test here into four 5-min bins. Mean (± SEM) lever presses per min across the test are shown in Figure 3*J*. From this figure, it is clear that both groups SHAM and mOFC initially responded in accordance with the punishment contingencies (unpunished > punished), but only group SHAM continued to respond this way for the duration of the test, whereas group mOFC began to respond equally on both levers (unpunished = punished) towards the end of the test. Indeed, there was a punishment × time interaction, *F*(2.144, 23) = 3.511, *P* = 0.035 (Greenhouse Geisser correction applied), and simple effects revealed a significant punishment × linear interaction for group mOFC, *F*(1, 23) = 5.652, *P* = 0.026, but not for group SHAM, *F* < 1. This suggests that the punishment effect was linearly altered across the test for group mOFC but not group SHAM.

To further determine the source of these effects, we examined the first 5 min and the last 5 min of this test separately. Mean (± 1 SEM) lever presses during the first 5 min are shown in Supplementary Figure 2*F*, and mean (±1 SEM) lever presses during the last 5 min are shown in Supplementary Figure 2*G*, with individual data points for each animal. In the first 5 min, both groups SHAM and mOFC responded in accordance with the punishment contingencies (unpunished > punished) because there was a main effect of punishment, *F*(1, 23) = 21.63, *P* = 0.00, which did not interact with group, *F* < 1. In contrast, performance during the last 5 min was impaired for group mOFC but not group SHAM, because there was a group × punishment interaction, *F*(1, 23) = 7.27, *P* = 0.013. Follow up simple effects reveal that this interaction consisted of a significant effect of punishment (unpunished > punished) for group SHAM, *F*(1, 23) = 13.62, *P* = 0.001, but not group mOFC (unpunished = punished), *F* < 1.

This result suggests that the prior test results were not an artifact of an underpowered test, or the contingency change from extinction to pellets-only. Rather, animals with mOFC lesions rapidly lost selective punishment avoidance across these tests, suggesting that extinction of the punishment contingencies was facilitated for group mOFC relative to group SHAM. Thus, these results present only partial support for our prediction that mOFC lesions would prevent the retrieval of punishment contingencies. Reasons for this divergence from our hypothesis are discussed below.

#### Pavlovian Fear Conditioning

##### Conditioned Suppression (During Presurgery Conditioned Punishment)

Mean (± SEM) conditioned suppression ratios to the CS+ and CS− over days of presurgery conditioned punishment training are shown in Figure 4*A* for group SHAM and Figure 4*B* for group mOFC. It is clear from these figures that animals in both groups acquired specific fear to the CS+ and not the CS− and did not differ in their acquisition. Statistical analysis revealed that there was no main effect of group, *F* < 1, but there was a main effect of CS, *F*(1, 23) = 267.75, *P* < 0.001, that did not interact with group, *F* < 1.

##### Conditioned Suppression (During Conditioned Punishment Retraining)

Mean (±SEM) conditioned suppression ratios to the CS+ and CS− during conditioned punishment retraining are shown in Figure 4*C* for group SHAM and Figure 4*D* for group mOFC. It is clear that both groups once again showed selective conditioned suppression to the CS+ than to the CS−, indicating intact Pavlovian fear for both groups. Statistical analysis reveals that there was no main effect of group, *F* < 1, but there was a main effect of CS, *F*(1, 23) = 387.63, *P* = < 0.001 that did not interact with group, *F* < 1.

## Discussion

Together, current results demonstrate a causal role for mOFC in instrumental punishment avoidance, but not Pavlovian fear conditioning. In Experiment 1, mOFC lesions prevented conditioned punishment learning. Sham controls demonstrated intact conditioned punishment learning by avoiding a punished lever that earned a CS+ and footshock whilst continuing to respond on an unpunished lever that earned a neutral CS−, whereas animals with mOFC lesions responded equally on punished and unpunished levers. This deficit was specific to instrumental punishment learning and not Pavlovian fear conditioning because Sham and mOFC groups both selectively suppressed lever-pressing during CS+ relative to CS− presentations. This previously-identified phenotype of punishment insensitivity has been attributed to failures in detecting lean punishment contingencies (Jean-Richard-dit-Bressel et al. 2019). Consistent with this, we found that doubling the frequency of outcomes (i.e., pellets, CS+, CS−, and footshock) allowed group mOFC to overcome their initial impairments and avoid the punished response in favor of the unpunished response.

Experiment 2 tested whether mOFC lesions also prevented the retrieval of conditioned punishment contingencies. For this experiment, lesions were administered after conditioned punishment training. Following recovery, animals were given a 5-min extinction test (i.e., no outcomes delivered) followed by a 10-min test in which both levers earned only pellets. In the 5-min extinction test, both groups demonstrated statistically intact punishment avoidance. However, only the SHAM group continued to respond in this manner in the subsequent pellet-only test, whereas mOFC-lesioned animals responded indiscriminately on both levers (i.e., unpunished = punished). Rats were subsequently retrained on conditioned punishment for 5 days, and then given a pellet-only test for 20 min. During retraining, both groups SHAM and mOFC were able to selectively respond to the unpunished lever over the punished lever, suggesting that mOFC-lesioned rats could relearn punishment contingencies. On the final pellet-only test, both groups once again initially responded according to the punishment contingencies (unpunished > punished), but only Sham animals did so throughout the entire test. Group mOFC, on the other hand, responded equally on both levers for the latter half of the test, replicating the pattern observed in prior tests. This suggests mOFC lesions may accelerate punishment extinction.

Together, these findings provide the first causal evidence that disruption of mOFC function reduces sensitivity to punishment. They provide only partial support, however, for our hypothesis that mOFC lesions would prevent animals from inferring unobservable aversive outcomes as the consequence of their actions. The results of Experiment 1 generally support this account because mOFC lesions initially prevented conditioned punishment learning, which is measured during the ITI when CSs and punishing footshocks had to be inferred. Moreover, when outcomes were doubled in frequency, thus reducing the need to infer outcomes from memory storage as they could be held in more recent working memory, mOFC-lesioned animals were able to overcome their initial impairment. Nevertheless, increased “observability” is not the only reason mOFC-lesioned animals may have overcome their initial punishment learning deficit, as it is possible that the doubling of outcomes simply made the punishment even more aversive, and/or made the task of knowing which lever to avoid easier. Moreover, the results of Experiment 2, are particularly difficult to reconcile with this account. During both sets of retrieval tests in Experiment 2, it appeared that group mOFC did initially retain and express the punishment contingencies (i.e., unpunished > punished), despite extinguishing them more quickly than group SHAM. Furthermore, reacquisition of conditioned punishment after mOFC lesion surgery was completely intact for group mOFC, despite again being measured during the ITI when outcomes had to be inferred. These effects suggest that, under some circumstances at least, group mOFC were able to infer punishing outcomes even when unobservable.

Taken together with our prior findings (Bradfield et al. 2015, 2018), current results suggest that the role for mOFC in inferring the unobservable outcomes of actions might be limited to appetitive outcomes. This would further imply that mOFC regulates instrumental aversive learning via a different psychological mechanism. One possibility is that the mOFC is important for the formation of forming complex, three-term associations between actions–stimuli–aversive outcomes, particularly when the presentation of all three elements of the associations together is sparse. If this is the case, one prediction that could be made is that in contrast to the “conditioned” (or secondary) punishment procedure employed here, inactivating mOFC would be expected to leave punishment learning intact if a simpler “primary” punishment procedure were employed in which the relation between lever press and footshock is not intercepted with a stimulus. This is a question we intend to address in our future research.

There is another possibility, however, that could reconcile our initial interpretation of mOFC function with current results. It has been noted that with extended training, animals can learn to avoid a punished action out of habit (Ostlund and Balleine 2008). Thus it is possible that during the 7 days of presurgery conditioned punishment training in Experiment 2 (i.e., 2 days more than in Experiment 1), animals learned to avoid the punished lever in a habitual manner. Our account clearly predicts that habit learning should be unaffected by mOFC lesions because the ability to infer outcomes does not contribute to habitual stimulus–response (S–R) associations. Punishment retraining in Experiment 2 could have simply reinstated this habit in group mOFC, causing their reacquisition to be intact relative to group SHAM. During retrieval tests, this habit (unpunished > punished) could likewise have been applied for both groups initially, but when this habit “failed” in the absence of any punishing outcomes, animals returned to goal-directed responding as is known to occur under extinction conditions (Dezfouli et al. 2014). For group SHAM, this meant continued avoidance of the punished lever based on the goal-directed inference that presses could earn the unobservable CS+ and footshock. For group mOFC however, who could not infer these outcomes, goal-directed responding would have been elicited on the basis of the observable pellet outcomes only, which were earned equally by each lever (unpunished = punished).

Another surprising finding from the current study was the lack of any behavioral differences between animals that received anterior versus posterior mOFC lesion assignment. As mentioned, we have previously found the anterior but not posterior mOFC to be particularly important for the inference of outcome representations (Bradfield et al. 2018), and thus expected that this anterior/posterior dichotomy to also apply to punishment learning. However, we did not find any support for this hypothesis. As we were unable to separate these lesions anatomically, due to a significant site of overlap (centered at +4.68 mm anterior to bregma), it is possible that all of our current lesions simply targeted the same functionally important mOFC region. Despite our inability to separate the contributions of the anterior versus posterior mOFC to punishment sensitivity, however, our results do suggest that the functionally important region of mOFC is unlikely to extend beyond its posterior limit to adjacent regions such as the prelimbic or infralimbic cortices (more recently referred to as anterior cingulate areas A32V, and A25, respectively; Paxinos and Watson 2014; Laubach et al. 2018). This is because the lesion spread observed in animals who were assigned to anterior mOFC placements did not approach either prelimbic or infralimbic cortex, with no anterior lesions in either experiment extending beyond +4.2 mm from bregma (see Figs 1*E* and 3*A* for anterior placements), yet, their behavior was identical to animals with more posterior mOFC placements who did experience some lesion overlap with these regions (see Figs 1*F* and 3*B* for placements, and Supplementary Figs 1*C–F* and 2*A–E* for behavioral comparisons). On this basis, therefore, it is much more likely that it was the damage to the mOFC in these animals, rather than damage to the prelimbic or infralimbic cortex in posterior animals only, that caused our observed effects.

The finding that mOFC lesions did not affect our measure of Pavlovian fear conditioning (conditioned suppression to the aversive CS+), although consistent with our hypothesis, does appear to be inconsistent with findings from two prior studies that have demonstrated a role for mOFC in Pavlovian fear conditioning (Rodriguez-Romaguera et al. 2015; Hsieh and Chang 2020). However, both these studies differ from the current study in several crucial ways. For instance, these prior studies measured freezing rather than conditioned suppression to index learning, making it possible that the mOFC specifically regulates fear conditioning as measured by freezing. We do find this somewhat unlikely, however, given that measures of freezing and conditioned suppression have been found to be highly correlated (Bouton and Bolles 1980) as well as having overlapping neural underpinnings, at least at the level of the amygdala (Lee et al. 2005). Further differences between prior and current studies include the fact that Hsieh and Chang (2020) pharmacologically activated mOFC, whereas we inactivated it. Rodriguez-Romaguera et al. (2015), on the other hand, found specific effects of mOFC inhibition on fear extinction rather than fear acquisition, and we did not test fear extinction in the current study. Therefore, either or any of these differences could account for the differential findings.

Although there is still much to be determined regarding mOFC’s regulation of punishment learning and retrieval, the current demonstration of a causal link is an important starting point, and one that has multiple implications. For instance, given the strong reciprocal links between mOFC and the basolateral amygdala (Hoover and Vertes 2011; Malvaez et al. 2019), and the central role of basolateral amygdala in punishment avoidance (Killcross et al. 1997), the mOFC-basolateral amygdala pathway is a prime candidate for the neural circuitry that underpins conditioned punishment. Medial OFC also has a number of other key connections that could mediate punishment learning, such as its strong outputs to nucleus accumbens core (Hoover and Vertes 2011), which is central to motivated behavior (Corbit et al. 2001; Corbit and Balleine 2011).

Moreover, it has now been demonstrated several times that the orbitofrontal cortex accommodates a functionally heterogeneous population of neuronal ensembles (Schoenbaum et al. 1999; Tremblay and Schultz 1999; Padoa-Schioppa and Assad 2006), some of which have been individually manipulated and demonstrated to regulate unique types of behavior (Jennings et al. 2019). Although most of these studies have focused on lateral OFC, it is possible that heterogeneous neuronal ensembles also exist within mOFC, and that their coordination is what allows mOFC to form complex, abstract outcome representations regardless of appetitive or aversive valence. The nonspecific excitotoxic lesions in the current study would have indiscriminately targeted all of these ensembles, but with the application of the increasingly specialized tools, future studies can begin to unravel the potential contributions of each with great precision. Furthermore, as lateral OFC inactivation has also been shown to affect primary punishment (Orsini et al. 2015; Jean-Richard-Dit-Bressel and McNally 2016; Verharen et al. 2019), similar studies of the lateral OFC’s contributions to conditioned punishment learning and expression will also be of great interest to future studies.

In conclusion, current results demonstrate a causal relationship between dysregulation of mOFC function and insensitivity to punishment but not Pavlovian fear conditioning. Further, mOFC activity appears to be particularly important for punishment learning when aversive outcomes are less frequently delivered. This could have a myriad of interesting clinical implications if translatable. For instance, it is possible that individuals suffering from compulsivity and insensitivity to punishment due to mOFC dysfunction might reinstate their sensitivity if punishing outcomes were delivered more frequently and/or consistently.

## Supplementary Material

Supplementary_material_for_submission_tgaa039

## Notes

We thank Gavan McNally for helpful discussions regarding these data. *Conflict of Interest:* None declared.
