# Supplementary material for: Medial Orbitofrontal Cortex Regulates Instrumental Conditioned Punishment, but not Pavlovian Conditioned Fear
Source: Cereb Cortex Commun. 2020 Jul 30;1(1):tgaa039. doi: 10.1093/texcom/tgaa039 (PMC8152850; doi:10.1093/texcom/tgaa039)
Supplement: Supplementary_material_for_submission_tgaa039 [file supplementary_material_for_submission_tgaa039.docx]

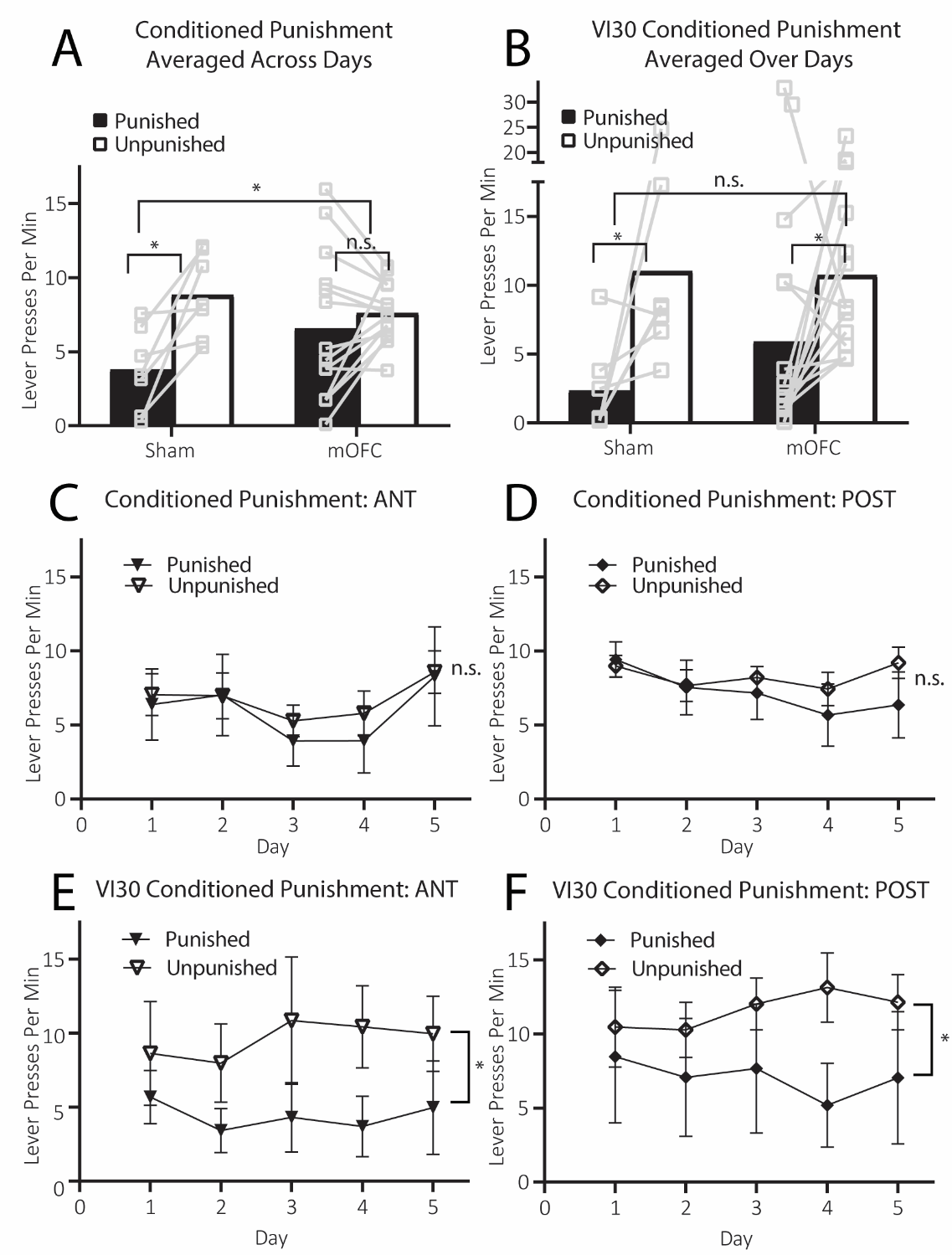
**Supplementary Figure 1. Supplementary experimental data for Figure 1 (Experiment 1).** A) Mean lever presses per min for initial conditioned punishment training. These are the same data shown in Figure 1H-I except averaged across days, and with individual data points shown for each animal, B) Mean lever presses per min for VI30 conditioned punishment training. These are the same data shown in Figure 1J-K except averaged across days, with individual data points shown for each animal, C) Mean (± 1 SEM) lever presses per min during initial conditioned punishment training graphed in accordance with animals assigned to receive anterior mOFC lesions, data extracted from Figure 1I D) Mean (± 1 SEM) lever presses per min during initial conditioned punishment training graphed in accordance with animals assigned to receive posterior mOFC lesions, data extracted from Figure 1I, E) Mean (± 1 SEM) lever presses per min during VI30 conditioned punishment training graphed in accordance with animals assigned to receive anterior mOFC lesions, data extracted from Figure 1K, F) Mean (± 1 SEM) lever presses per min during VI30 conditioned punishment training graphed in accordance with animals assigned to receive posterior mOFC lesions, data extracted from Figure 1K. * = p < .05, n.s. = non-significant, p > .05.


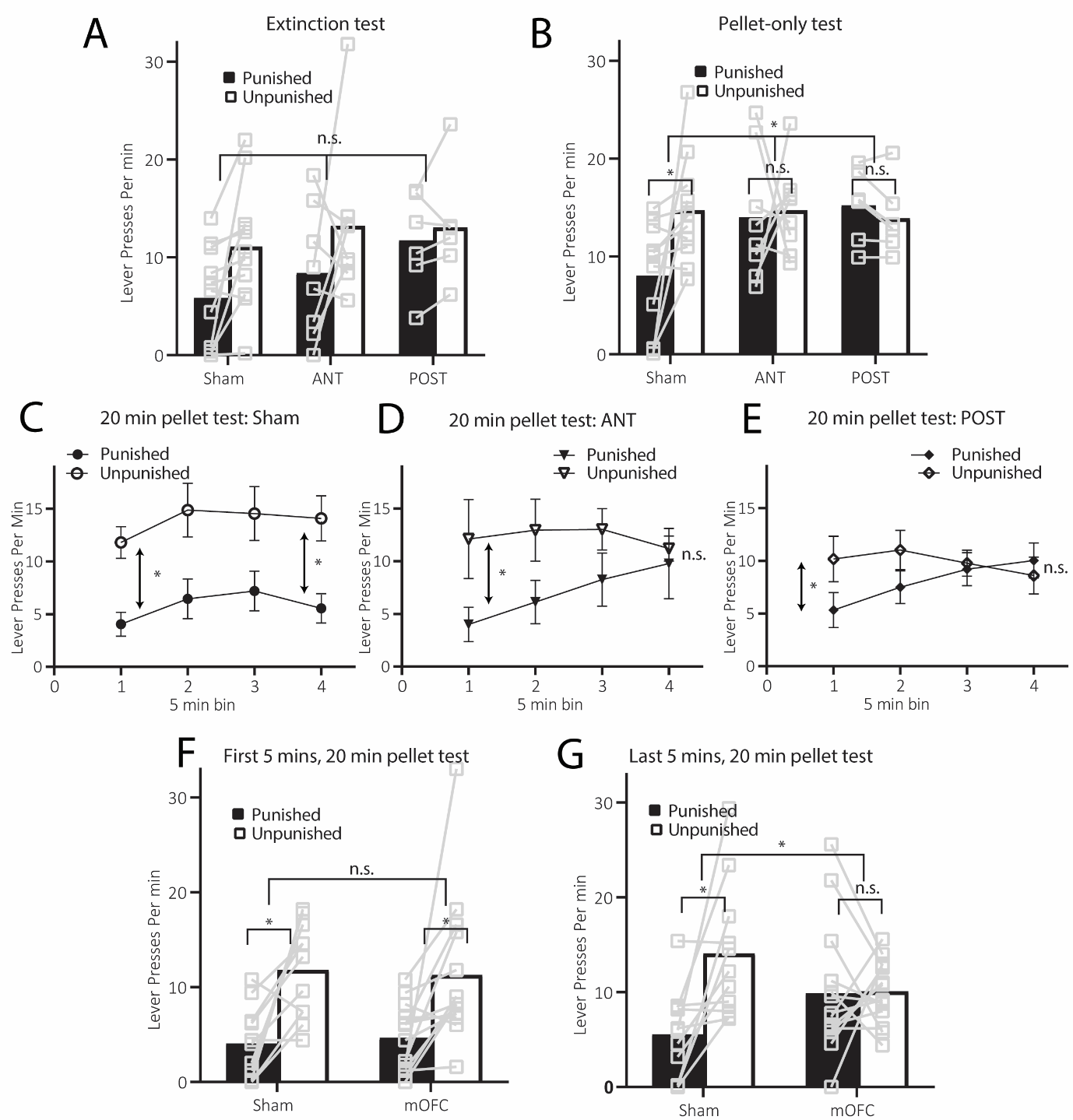


**Supplementary Figure 2. Supplementary experimental data for Figure 3 (Experiment 2).** A) Mean lever presses per min for the 5 min extinction test, graphed in accordance with animals assigned to receive anterior and posterior mOFC lesions, respectively, data extracted from Figure 3F, B) Mean lever presses per min for the 10 min pellet-only test, graphed in accordance with animals assigned to receive anterior and posterior mOFC lesions respectively, data extracted from Figure 3G, C) Mean (± 1 SEM) lever presses per min for the 20 min pellet-only test for group Sham, data extracted from Figure 3J, D) Mean (± 1 SEM) lever presses per min for the 20 min pellet-only test graphed in accordance with animals assigned to receive anterior mOFC lesions, data extracted from Figure 3J, E) Mean (± 1 SEM) lever presses per min for the 20 min pellet-only test graphed in accordance with animals assigned to receive posterior mOFC lesions, data extracted from Figure 3J, F) Mean lever presses per min for the first 5 mins of the 20 min pellet-only test, with individual data points for each animal, data extracted from Figure 3J G) Mean lever presses per min for the last 5 mins of the 20 min pellet-only test, with individual data points for each animal, data extracted from Figure 3J. * = p < .05, n.s. = non-significant, p > .05.
